# Assessment of Distinct Electrophysiological Parameters in Rectal Biopsies for the Choice of the Best Diagnosis/Prognosis Biomarkers for Cystic Fibrosis

**DOI:** 10.3389/fphys.2020.604580

**Published:** 2020-12-23

**Authors:** Iris A. L. Silva, Aires Duarte, Fernando A. L. Marson, Raquel Centeio, Tereza Doušová, Karl Kunzelmann, Margarida D. Amaral

**Affiliations:** ^1^BioISI – Biosystems and Integrative Sciences Institute, Faculty of Sciences, University of Lisbon, Lisbon, Portugal; ^2^Laboratory of Cell and Molecular Tumor Biology and Bioactive Compounds, São Francisco University, Bragança Paulista, Brazil; ^3^Laboratory of Human and Medical Genetics, São Francisco University, Bragança Paulista, Brazil; ^4^Institut für Physiologie, Universität Regensburg, Regensburg, Germany; ^5^Department of Paediatrics, 2nd Faculty of Medicine, Motol University Hospital, Charles University, Prague, Czechia

**Keywords:** CFTR, Ussing chamber measurements, disease severity, genotype-phenotype correlations, CFSPID

## Abstract

Most cases of Cystic Fibrosis (CF) are diagnosed early in life. However, people with atypical CF forms pose diagnosis dilemmas, requiring laboratory support for diagnosis confirmation/exclusion. *Ex vivo* analysis of fresh rectal biopsies by Ussing chamber has been the best discriminant biomarker for CF diagnosis/prognosis so far. Here we aimed to evaluate different electrophysiological parameters from Ussing chamber analysis of rectal biopsies from people with CF (PwCF) to establish the one with highest correlations with clinical features as the best CF diagnosis/prognosis biomarker. We analyzed measurements of CFTR-mediated Cl^–^ secretion in rectal biopsies from 143 individuals (∼592 biopsies), the largest cohort so far analyzed by this approach. New parameters were analyzed and compared with the previous biomarker, i.e., the IBMX (I)/Forskolin (F)/Carbachol (C)-stimulated short-circuit current (I’_sc–I/F/C_). Correlations with clinical features showed that the best parameter corresponded to voltage measurements of the I/F + (I/F/CCH) response (V_I/F+I/F/C_), with higher correlations vs. I’_sc–I/F/C_ for: sweat chloride (59 vs. 52%), fecal elastase (69 vs. 55%) and lung function, measured by FEV_1_ (27 vs. 20%). Altogether data show that V_I/F+I/F/C_ is the most sensitive, reproducible, and robust predictive biomarker for CF diagnosis/prognosis effectively discriminating classical, atypical CF and non-CF groups.

## Introduction

Cystic fibrosis (CF; OMIM: #219700) is a lethal inherited disease caused by pathogenic variants in the CF transmembrane conductance regulator (*CFTR*) gene, resulting in impairment of CFTR protein expression, function and/or stability (De Boeck and Amaral, 2016). CFTR is a cAMP-dependent, phosphorylation-activated anion channel that transports chloride (Cl^–^) and bicarbonate (HCO_3_^–^) across the apical plasma membrane of epithelial cells (De Boeck and Amaral, 2016). Absence of functional CFTR protein leads to impaired transepithelial balance of ions and fluid in epithelial cells lining the airways, intestine, sweat gland, and pancreas, among other organs. Although CF is a multi-organ disease, the respiratory disorder represents the major cause of morbidity and mortality. The accumulation of mucus in the airways, chronic inflammation, and recurrent bacterial infections lead to progressive deterioration of lung function, ultimately resulting in respiratory failure (Lopes-Pacheco, 2020). For most people with CF (PwCF), the diagnosis is usually established early in life either by newborn screening or by clinical characteristic features (Marcão et al., 2018). Nevertheless, establishment of a definite CF diagnosis requires evidence of CFTR dysfunction, most commonly through two positive sweat Cl^–^ tests and/or identification in both alleles of *CFTR* variants defined as disease-causing (Castellania et al., 2008). However, diagnosis of individuals with atypical forms of CF may pose dilemmas, as these individuals may present normal or borderline levels of Cl^–^ in the sweat, without identification of two known pathogenic variants (Levy and Farrell, 2015; Castellani et al., 2019). Moreover, the implementation of robust neonatal screening programs has led to the identification of increasing numbers of still asymptomatic CF screen positive with inconclusive diagnosis (CFSPID) babies (Barben and Southern, 2016), posing new challenges to the CF diagnosis paradigm and requiring new assays for CF confirmation. In these cases, further laboratory analyses are mandatory to confirm/exclude a CF diagnosis, including transepithelial nasal potential difference (NPD) measurements or *ex vivo* CFTR functional assessment in rectal biopsies (Sousa et al., 2012; Beekman et al., 2014; Bell et al., 2015). The latter was indeed demonstrated to be the most sensitive test for the diagnosis and prognosis of CF, showing the best correlations with clinical parameters, including age-stratified lung function (Sousa et al., 2012).

Here we aimed to evaluate different electrophysiological parameters obtained from Ussing chamber analysis of CFTR-mediated Cl^–^ secretion in rectal biopsies from 143 individuals (∼592 biopsies), the largest cohort so far analyzed by this approach. Our goal is to establish the Ussing chamber parameter that best correlates with clinical features to be used as reference CF diagnosis/prognosis biomarker. Our analyses and comparison with the previously used biomarker, i.e., the IBMX (I)/Forskolin (F)/Carbachol (C)-stimulated equivalent short-circuit current (I’_sc–I/F/C_), calculated by Ohm’s law from measured voltage, reveal that a new parameter – voltage measurements of the combined I/F + (I/F/CCH) response (V_I/F+I/F/C_) – evidenced the best correlations with clinical features for: sweat Cl^–^, fecal elastase, lung function, body mass index and presence of bacterial colonization.

## Materials and Methods

### Cohort and Sample Selection

All subjects (or parents/tutors, for those <18 years) gave their informed consent for inclusion before they participated in the study. The study was conducted in accordance with the Declaration of Helsinki, and the protocol was approved by the Ethics Committee of Research, Ethics Committee of the Faculty of Medical Sciences, University of Campinas, as well as of the Santa Maria Hospital, Lisbon, Portugal and the Paediatrics Department of Motol University Hospital, Prague, Czechia. Altogether ∼592 freshly excised rectal biopsies were analyzed from 143 individuals from Portugal, Czechia, Spain, Turkey, United Arab Emirates and Brazil, including CF patients with confirmed diagnosis [e.g., by newborn screening and/or by gene sequencing (Supplementary Table 1)], PwCF clinical suspicion [e.g., due to high sweat chloride (SwCl) concentrations and/or pancreatic impairment] and age-matched non-CF controls. Individuals from Spain, Turkey and the United Arab Emirates were biopsied in Lisbon. Some “non-CF” individuals were also genotyped (Supplementary Table 1) because they were suspicious cases of CF, however, other “non-CF” individuals were just healthy volunteers for the biopsy protocol, and therefore were not genotyped. All CF centres involved followed the same SOP from Farrell et al. (2017) and sweat tests were performed using the Gibson and Cooke method using standardized procedures. All the CF centres involved use the same method for more than 20 years and in all the sweat test was performed by experienced nurses/technicians.

### Rectal Biopsies Procedure

Colon preparation (cleaning) was done by applying an enema of saline solution (0.9% NaCl) on the biopsy day, and glycerol suppository rectally on the day before. A total of 6–8 superficial rectal mucosa specimens (3–4 mm in diameter) were obtained without sedation by colon forceps with visual examination, avoiding the risk of bleeding or of collecting damaged tissue, and immediately stored in ice-cold Advanced DMEM F12 medium (Invitrogen) with 5% (v/v) Fetal Bovine Serum (FBS, Invitrogen).

### Measurement of CFTR-Mediated Cl^–^ Secretion in Ussing Chamber

**FIGURE 1:**
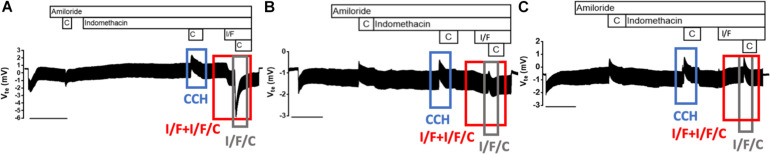
Representative original tracings from Ussing chamber measurements of CFTR-mediated Cl^–^ secretion in rectal biopsies from 143 individuals with three distinctive patterns. Effects of cholinergic stimulation by carbachol (CCH, 100 μM, basolateral, blue box), cAMP-dependent by IBMX/Forskolin (I/F, 100 μM/2 μM, basolateral), and I/F/C combined on transepithelial voltage (V_te_) represented by the red box, on rectal biopsies from: **(A)** non-CF individuals, **(B)** individuals with atypical CF, and **(C)** individuals with classical CF. The gray box represents the effect of the last I/F/C response. Experiments were performed in the presence of Amiloride (Amil, 20 μM, luminal) and/or Indomethacin (Indo, 10 μM, basolateral), as indicated above tracings.

Rectal biopsy specimens (3–5 per patient) were mounted and analyzed in modified perfused micro-Ussing chambers as previously described under open-circuit conditions (Mall et al., 2004; Sousa et al., 2012). Briefly, the luminal and basolateral surfaces of the epithelium were continuously perfused (5 mL/min) with Ringer solution with the following composition (mmol/L): NaCl 145, KH_2_PO_4_ 0.4, K_2_HPO_4_ 1.6, D-glucose 5, MgCl_2_ 1, Ca-gluconate 1.3, pH 7.40, at 37°C. Tissues were equilibrated in the micro-Ussing chambers for 30 min in perfused Ringer solution before measurements. Amiloride (Amil, 20 μM, luminal) was added to block electrogenic sodium (Na^+^) absorption through the epithelial Na^+^ channel (ENaC). Indomethacin (Indo, 10 μM, basolateral) was applied for 20–40 min to inhibit endogenous cAMP formation through prostaglandins. cAMP-dependent and cholinergic Cl^–^ secretion in human rectal tissues relies on functional CFTR. Thus, we used 3-isobutyl-1-methylxantine (IBMX/I, 100 μM, basolateral) and forskolin (F, 2 μM, basolateral) to activate cAMP-dependent Cl^–^ secretion and carbachol (CCH, 100 μM, basolateral) for cholinergic co-activation. In this protocol, Amil was constantly perfused in the luminal side during the experiment. In the basolateral side the sequential addition of compounds was: 1. CCH- > Indo, 2. Indo + CCH, 3. Indo, 4. Indo + IBMX + F, 5. Indo + IBMX + F + CCH, as depicted in Figure 1. Transepithelial resistance (R_te_) was determined by applying intermittent (1 s) current pulses (ΔI = 0.5 μA), and the corresponding changes in Vte (ΔVte), as well as basal Vte are recorded continuously during the course of the experiment. Values for the Vte are referred to the serosal side of the epithelium. The equivalent short-circuit current (I_eq–sc_) was calculated according to Ohm’s law (I_sc_ = V_te_/R_te_), after appropriate correction for fluid resistance and after subtracting the resistance of the empty chamber. Quality criteria, such as basal negative transepithelial V_te_ in the beginning of the experiments and tissue R_te_ were taken in to account. When not present, the biopsies were rejected and excluded from the analysis (∼28 biopsies were rejected, out of a total of 592 analyzed, i.e., ∼5%). Each individual value reflects the mean of all biopsies analyzed for that individual.

### Statistical Analyses

All statistical analyses were computed in R version 3.6.3. Biopsy parameters were evaluated with Turkey’s method to identify outliers ranged above and below 1.5^∗^IQR. Normality of data distribution was assessed with Shapiro–Wilk test. Clinical parameters were compared between the groups by One-Way ANOVA test or Kruskal–Wallis Rank Sum test, when data were not normal distributed (R Core Team, 2020). The correlations between biopsy and clinical parameters were analyzed with Pearson’s or Spearman’s correlation coefficient. The correlations between the electrophysiological parameters and forced expiratory volume in the first second (FEV1) and forced vital capacity (FVC) were adjusted to the age at study. This partial correlation was analyzed with Pearson’s correlation coefficient for data with normal distribution or Spearman’s correlation coefficient for data not normal distributed (Kim, 2015; Wickham, 2016; Harrell, 2020).

## Results

### Overview of the Clinical Features in the Cohort

The cohort under study here was composed by PwCF and non-CF individuals (as controls) from Portugal, Brazil, Czechia, Spain, Turkey, and United Arab Emirates, in a total of 143 participants (Supplementary Tables 1, 2).

The diagnosis of CF by each physician was established based on international criteria (Farrell et al., 2008), i.e., the presence of characteristic clinical features, such as lung disease and/or gastrointestinal symptoms, and evidence of CFTR dysfunction, either by a two positive SwCl tests (≥60 mEq/L) and/or the identification of two CF-causing mutations. The functional classification of these mutations was also used to define mutation severity. Using these criteria three different diagnosis groups were *a priori* defined, namely (Supplementary Table 2): (1) firstly, classical CF, including PwCF presenting a severe CF phenotype, with lung impairment, high SwCl concentration (≥60 mEq/l), pancreatic insufficiency (PI) and two severe CF-causing mutations, i.e., in classes I, II, III or VII (Hirtz et al., 2004; De Boeck and Amaral, 2016); (2) secondly, atypical CF, including PwCF with later onset of the disease, borderline SwCl concentration, less respiratory involvement, pancreatic sufficiency (PS), CFTR mutations associated with residual function [i.e., in classes IV, VI or VI (De Boeck and Amaral, 2016) and classified as such either in CFTR2 database^1^ or in functional studies], or considered CFSPID; (3) thirdly, non-CF individuals, including healthy individuals used here as controls. The non-CF group also included individuals with suspicion of CF (due to high SwCl tests, or PI), but that were considered non-CF after the analysis of CFTR function in rectal biopsies. Overall, no CFTR mutations were found in the non-CF group. Unexpectedly high SwCl tests could be due to medication, eczema, malnutrition, metabolic, endocrine or skin diseases, or other factors (Guglani et al., 2015).

To assess disease severity, several clinical markers associated with CF were also evaluated: presence of bacterial colonization, namely Pseudomonas aeruginosa (Pa); determination of fecal elastase E1 (FE-1) concentration to assess exocrine pancreatic function; measurements of sweat chloride (SwCl) concentration, the key CF biomarker; body mass index (BMI); and assessment of the forced expiratory volume in the first second (FEV_1_), as well as forced vital capacity (FVC), as lung function readouts. The frequencies of each clinical parameter for individuals in the cohort are listed in Supplementary Table 2, and the mean values for each diagnosis group are in Supplementary Table 3. In summary, the results show that the mean SwCl in the classical CF group are significantly higher than in the other two groups, which indicates that SwCl is in fact a good biomarker to assess disease severity (Supplementary Table 3). However, some individuals without CF have high SwCl values (mean of non-CF in the cohort is 53 mEq/L), and indeed false positive SwCl tests have been described in the literature and thus possibly affecting the expected values for non-CF individuals (Mishra et al., 2005; Guglani et al., 2015). Furthermore, some individuals in the non-CF group had a suspected CF diagnosis precisely due to abnormal SwCl values (although latter shown to not have CF), and therefore inflating SwCl values in this group. Our results also show that the atypical CF group displays intermediate SwCl values, but on average higher than 30 mEq/L, the cut-off to discard CF (Accurso et al., 2014). FE-1 was also considerably different among groups, with significantly lower levels in the classical CF group, when compared to the atypical CF and non-CF groups, highlighting the high prevalence of PI among PwCF in this group (Supplementary Table 3).

### Assessment of CFTR-Mediated Cl^–^ Secretion in Rectal Biopsies

The main goal of this work was to find the best predictive biomarker from analysis of electrophysiological measurements in rectal biopsies Ussing chamber that could be used both as reference for CF diagnosis and prognosis. Measurements of CFTR-mediated Cl^–^ secretion in rectal biopsies of PwCF were previously shown to be the best discriminator of CF diagnosis and prognosis using values of I/F/CCH-stimulated equivalent short-circuit current (I’_sc–I/F/C_) determined by Ohm’s law from measured transepithelial voltage (V_te_) (Mall et al., 2000, 2004; Hug et al., 2011; Sousa et al., 2012). Here we analyzed these data by testing different electrophysiological parameters so as to determine the one with the best correlations with clinical parameters using the largest (143 individuals, ∼592 biopsies) and most diverse cohort of PwCF analyzed so far by this approach.

As previously established (Hirtz et al., 2004), our results show three different tracing patterns depending on the amount of active CFTR present in the rectal biopsies (Figure 1): (1) a negative voltage deflection upon the first CCH stimulation, as well as a negative response to I/F which is further enhanced by CCH addition in the presence of I/F – I/F/C – reflecting normal CFTR activity and Cl^–^ secretion thus indicative of absence of CF; (2) a positive voltage peak after the first CCH stimulation, which indicates CFTR impairment, followed by a biphasic (positive and negative) response to I/F and I/F/C, indicating presence of residual CFTR activity; (3) classical CF, which is characterized by three positive voltage peaks, upon CCH or I/F/C stimulation, showing total absence of CFTR activity and consistent with classical forms of CF (Figure 1).

From these Ussing chamber tracings, distinct parameters can be extracted and to date the gold standard one, previously correlated with other CF biomarkers and clinical data, was the equivalent short-circuit current of the I/F/C response (I’_sc–I/F/C_), obtained from the correspondent measured V_te_ response by Ohm’s law (Sousa et al., 2012). In the present work, we compared this parameter with additional electrophysiological ones that could be extracted from the Ussing chamber measurements and correlated them with several clinical parameters in a large cohort of PwCF including individuals from other countries for greater diversity.

### Comparative Analysis of I’_sc–I/F/C_ With New Electrophysiological Parameters

Several electrophysiological parameters were evaluated for their correlation with different clinical features. These included (see Figure 1 and Table 1):

1. The voltage peak resulting from the second CCH stimulation (under indomethacin), that represents the solo effect of the cholinergic secretion of basolateral potassium (K^+^): that we termed V_CCH_;
2. The sum of the voltage peaks resulting from the combined I/F and I/F/CCH responses: termed V_I/F+I/F/C_;
3. The voltage peak resulting from the third CCH stimulation (under I/F): termed V_I/F/C_;
4. The difference between V_CCH_ and V_I/F+I/F/C_ responses, normalized to V_CCH_: [V_CCH_-V_I/F+I/F/C_]/V_CCH_;
5. The difference between V_CCH_ and the V_I/F/C_ responses, normalized to V_CCH_: [V_CCH_-V_I/F/C_]/V_CCH_.

**TABLE 1:**
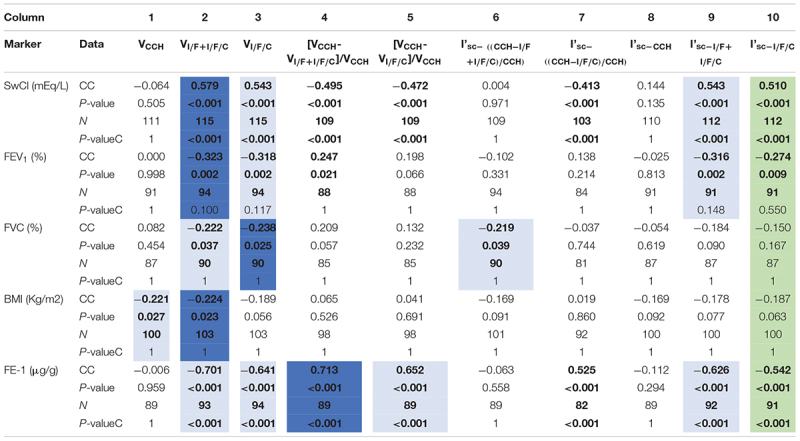
Correlation of electrophysiological parameters obtained from Ussing chamber measurements in rectal biopsies with CF biomarkers and clinical CF features.

*Positive p-values are shown as bold (p-values < 0.05 were considered significant). In green is shown the standard (previously validated) parameter for Ussing chamber analysis of rectal biopsies (Hirtz et al., 2004; Sousa et al., 2012). In blue are the parameters evidencing higher correlations with the clinical markers related with CF, being color intensity (dark or light) associated with the value of the correlation coefficient (CC). The Spearman correlation was done in all cases except for the spirometry markers (FEV_1_ and FVC) where a partial age-stratified correlation was used. P-valueC represents the p-value after Bonferroni correction. BMI, body mass index; FEV1, forced expiratory volume in the first second; FVC, forced vital capacity; FE-1, fecal elastase.*

Transepithelial voltage (V_te_) measurements for the above parameters were directly obtained from the Ussing chamber tracings (Table 1, columns 1–5). Similar parameters for the corresponding equivalent short-circuit currents (I_eq–sc_, calculated by Ohm’s law) were also assessed (Table 1, columns 6–10). All these electrophysiological parameters were correlated with the CF biomarkers and clinical features mentioned before, including SwCl, FEV_1_, FVC, FE-1, and BMI. Correlation results (Table 1) showed a significant correlation of I’_sc–I/F/C_ (column 10) with SwCl, FEV_1_ and FE-1, which was expected since this was the previously validated biomarker (Hirtz et al., 2004; Sousa et al., 2012).

Regarding the novel parameters tested, the one evidencing the highest and most significant correlations with SwCl, FEV_1_, FVC, BMI and FE-1, was V_I/F+I/F/C_ (column 2), even when compared to the previously validated parameter I’_sc–I/F/C_. These data show that this novel parameter is more representative of the clinical state associated with CF (Table 1), thus being a better biomarker to predict disease liability. Furthermore, these results show that the sum of voltages resulting from the I/F and I/F/CCH responses (I/F + I/F/C), that correspond to the maximal activation of CFTR, has better correlations not only than I’_sc–I/F/C_, but also than the voltage correspondent to this I_eq–sc_, i.e., V_I/F/C_ (column 3) resulting from the last I/F/CCH response alone.

The correlations of this novel parameter – V_I/F+I/F/C_ – with each of the several CF biomarkers and clinical CF features analyzed are shown in detail in the next sections.

### Correlation of V_I/F+I/F/C_ With CF Disease Severity

**FIGURE 2:**
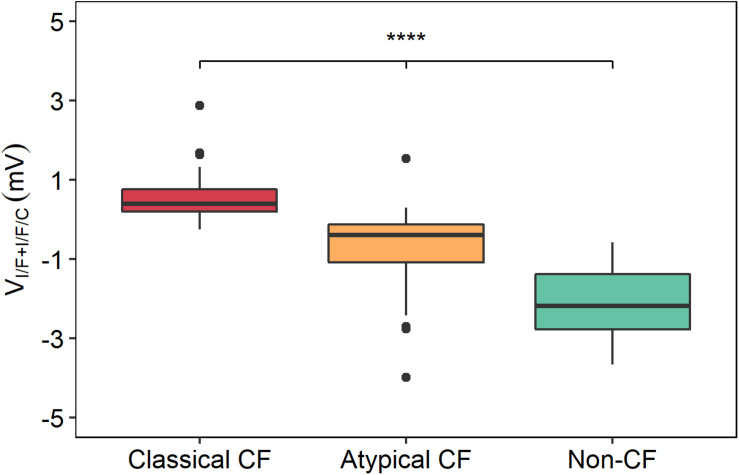
Distribution of V_I/F+I/F/C_ values between the three groups of individuals with distinct levels of disease severity. The V_I/F+I/F/C_ values were plotted for each group of individuals with distinct levels of disease severity, showing that the most severe disease state (classical CF) had higher V_I/F+I/F/C_ values, the atypical forms of CF had intermediate V_I/F+I/F/C_ values and non-CF controls had lower V_I/F+I/F/C_ values. Significant differences were found between these three groups by a Kruskal–Wallis rank sum test. (****p ≤ 0.0001).

Plotting of V_I/F+I/F/C_ values for the three groups (classical CF, atypical CF, and non-CF) evidences a clear distinction among these groups (Figure 2), where more negative values of V_I/F+I/F/C_ are associated with non-CF controls, intermediate values (i.e., residual CFTR activity) to PwCF with atypical disease, and higher V_I/F+I/F/C_ values (i.e., absence of functional CFTR) correspond to PwCF with classical CF forms.

#### Correlation of V_I/F+I/F/C_ With Sweat Cl^–^

**FIGURE 3:**
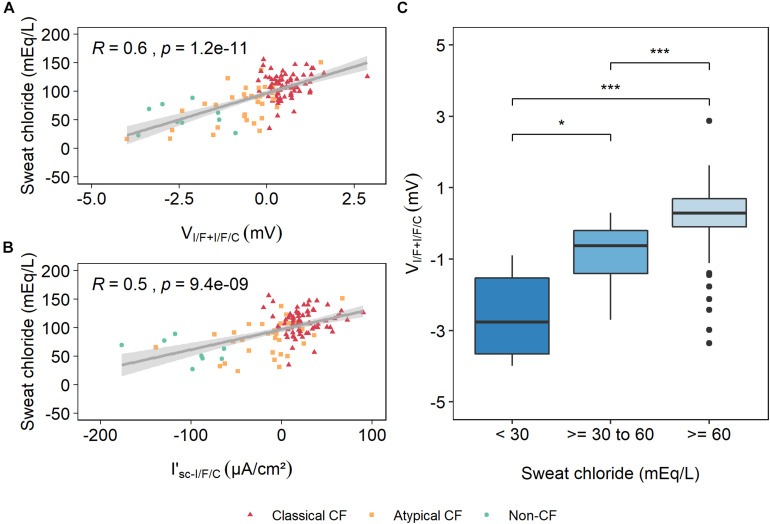
Correlation of sweat chloride (SwCl) concentration with V_I/F+I/F/C_ and I’_sc–I/F/C_. A Spearman rho rank correlation analysis showed that there is a strong and positive correlation between SwCl and V_I/F+I/F/C_ with a correlation coefficient of ∼0.6 **(A)**, whereas I’_sc–I/F/C_ evidenced a moderate correlation with a coefficient of ∼0.5 **(B)**. R represents the correlation coefficient and p is the *p*-value. **(C)** V_I/F+I/F/C_ values were plotted against SwCl values for each group of individuals with distinct levels of disease severity, showing that individuals with higher V_I/F+I/F/C_ values are associated with higher SwCl values, whereas individuals with lower V_I/F+I/F/C_ values are associated with lower SwCl values. Significant differences were found between these three groups by a Kruskal–Wallis rank sum test. (*p ≤ 0.05; **p ≤ 0.01). After Bonferroni correction only the 30–60 mmol/L and ≥60 mmol/L groups were significantly different (*p < 0.05; **p ≤ 0.01).

Elevated SwCl concentration is the gold standard biomarker for the diagnosis of CF (Schmidt and Sharma, 2020). Our analysis showed that the new electrophysiological parameter V_I/F+I/F/C_ has the best correlation with SwCl (Table 1), even higher than the previously validated parameter (I’_sc–I/F/C_): ∼58 vs. ∼51%, respectively (Figures 3A,B).

Data in Figure 3 show that low values of V_I/F+I/F/C_, corresponding to residual or normal Cl^–^ transport, correlate with the lowest SwCl values in the CF range (i.e., ≥39 mEq/L), whereas high values of V_I/F+I/F/C_ are associated with the highest levels of SwCl, also characteristic of individuals with classical forms of CF (Figure 3C). These results strongly suggest that V_I/F+I/F/C_, rather than I’_sc–I/F/C_, is a better predictor of disease severity and is highly correlated with SwCl values.

#### Correlation of V_I/F+I/F/C_ With Fecal Elastase E1

**FIGURE 4:**
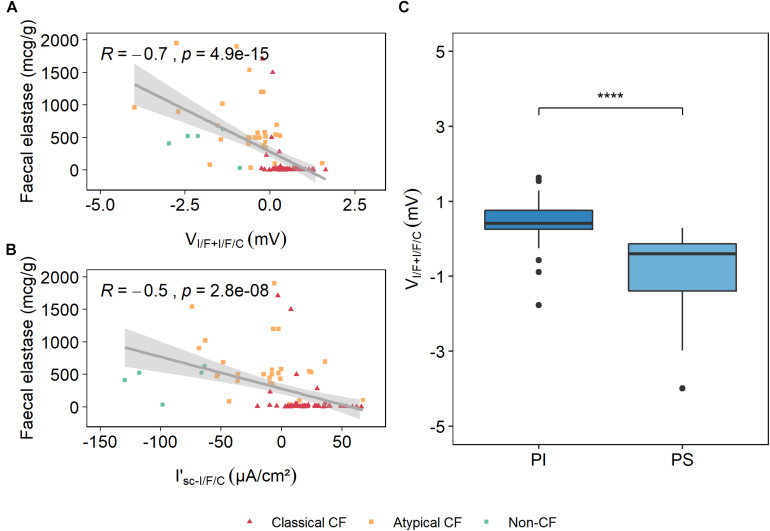
Correlation of fecal elastase E1 (FE-1) values and V_I/F+I/F/C_ and I’_sc–I/F/C_. Spearman rho rank correlation analysis showed that there is a strong negative correlation between FE-1 and V_I/F+I/F/C_, with a correlation coefficient of ∼0.7 **(A)**, whereas FE-1 correlation with I’_sc–I/F/C_ is moderate, with a coefficient of ∼0.5. **(B)** R represents the correlation coefficient and p represents the *p* value. **(C)** The values from the voltage of the maximal activation of CFTR measured in the I’_sc–I/F/C_ responses were plotted and grouped by the exocrine pancreatic function (PI and PS), and show that PwCF who are PI are associated with higher V_I/F+I/F/C_ values and that PS PwCF are associated with lower V_I/F+I/F/C_ values. [Kruskal–Wallis rank sum test (****p ≤ 0.0001)].

A cut-off value of 200 μg/g for FE-1 has shown a 98.7% sensitivity in detecting PwCF who are PI (Daftary et al., 2006), and therefore determination of FE-1 concentration is a simple and reliable method to assess exocrine pancreatic function in PwCF. Correlation of V_I/F+I/F/C_ with FE-1 has shown a negative correlation of 70% (Table 1), while the previously validated I’_sc–I/F/C_ had a correlation of ∼54% (Figures 4A,B).

Data in Figure 4 show that lower values of V_I/F+I/F/C_, associated with residual or normal Cl^–^ transport, correlate with higher levels of FE-1 (and PS), which also indicate the presence of residual CFTR activity. In contrast, higher values of V_I/F+I/F/C_, present in individuals with classical CF forms (Figure 1), are associated with lower levels of FE-1 (and PI), consistent with absence of functional CFTR (Figure 4C). These results clearly suggest that V_I/F+I/F/C_ is a robust parameter to predict exocrine pancreatic function.

#### Correlation of V_I/F+I/F/C_ With FEV_1_ and FVC

**FIGURE 5:**
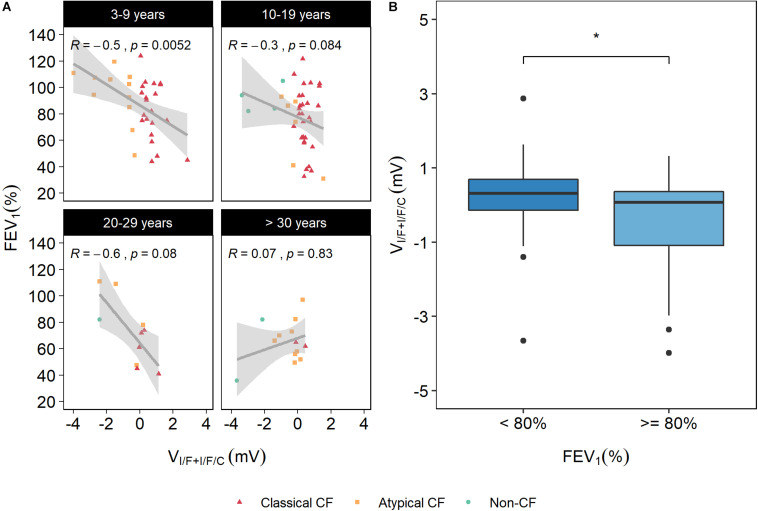
Correlation between age-stratified forced expiratory volume in 1 s [FEV_1_ (%)] and V_I/F+I/F/C_. **(A)** FEV_1_ distributed by age groups show a significant correlation of 50% between FEV_1_ and V_I/F+I/F/C_ for the 3–9 years sub-group. **(B)** Distribution of V_I/F+I/F/C_ values in individuals with FEV_1_ < 80% and ≥80% show that those with worst pulmonary function also have higher values of V_I/F+I/F/C_, whereas individuals with better pulmonary function have lower V_I/F+I/F/C_ values (i.e., higher CFTR function). Significant differences were found between these two groups by a Kruskal–Wallis rank sum test (*p ≤ 0.05).

Two lung function parameters (FEV_1_ and FVC) were correlated with the electrophysiological parameters under analysis, but no significant correlations were found (data not shown, Supplementary Figure 1). However, lung function has been shown to undergo distinct phases, going from a growth phase (from birth to early adulthood), a plateau phase, and a decline phase for FEV_1_ as a result of physiological lung aging (Agusti and Faner, 2019). In CF decline phase for FEV_1_ is particularly accentuated and rapidly accelerated by age (Schaedel et al., 2002). Therefore, we performed a partial correlation, i.e., stratified by age groups to have a better standardization regarding the relatively rapid decline of lung function in CF. The results showed a significant correlation (32%) between age-stratified FEV_1_ and V_I/F+I/F/C_, whereas the previously validated parameter I’_sc–I/F/C_ displayed a significant correlation of 27%. However, when different age sub-groups were analyzed a stronger significant correlation was found (∼50%) for the 3–9 years sub-group (Figure 5A), emphasizing the relevance of rectal biopsies measurements as predictive tool also for lung function in this group.

These results strongly suggest that V_I/F+I/F/C_ is a good predictor of lung function, at least for the lower age group (3–9 years). In general, data also show that individuals with lower FEV_1_ have higher V_I/F+I/F/C_ values, i.e., lower CFTR function, and that individuals with higher FEV_1_ have lower V_I/F+I/F/C_ values, i.e., residual CFTR activity (Figure 5B). Regarding FVC, the results showed a significant correlation (22%) between age-stratified FVC and V_I/F+I/F/C_, whereas the previously validated parameter I’_sc–I/F/C_ displayed a non-significant correlation (Table 1). These results highlight the robustness of this new parameter.

#### Correlation of V_I/F+I/F/C_ With Body Mass Index (BMI)

**FIGURE 6:**
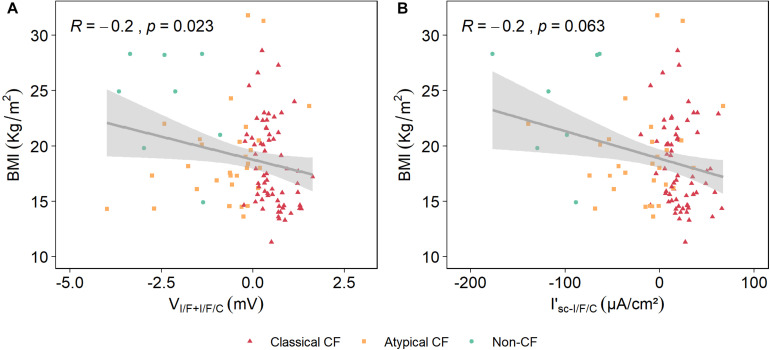
Correlation of body mass index (BMI) with V_I/F+I/F/C_ and I’_sc–I/F/C_. A Spearman correlation analysis showed that the correlation between BMI and V_I/F+I/F/C_ is 24% **(A)**, and that for I’_sc–I/F/C_ is 20% **(B)**.

Lower BMIs in PwCF are associated with worst lung function and disease prognosis, and therefore BMI correlation with V_I/F+I/F/C_ was also assessed here. Results show that there is a correlation between BMI and V_I/F+I/F/C_ being this of 22%, which albeit low is significant, whereas the previously validated parameter I’_sc–I/F/C_ displayed a non-significant correlation (Table 1 and Figure 6).

#### Correlation of V_I/F+I/F/C_ With Bacterial Colonization

**FIGURE 7:**
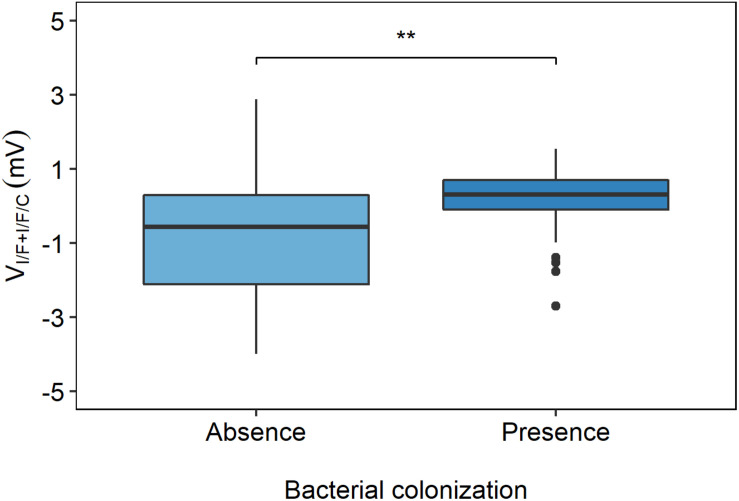
Distribution of V_I/F+I/F/C_ values in individuals with and without bacterial colonization. The V_I/F+I/F/C_ values were plotted and grouped by the absence or presence of bacterial colonization, and show that less bacterial colonization occurs in PwCF with lower V_I/F+I/F/C_ values and that higher frequency of bacterial colonization occurs in PwCF with higher V_I/F+I/F/C_ values, [Kruskal–Wallis rank sum test (**p ≤ 0.01)].

Bacterial colonization was assessed at the time of the study and included *Pseudomonas aeruginosa* (more frequent in older individuals), *Staphylococcus aureus* (more frequent in infants), *Burkholderia cenocepacia*, and more rarely *Escherichia coli*. Data in Figure 7 show that less bacteria colonization occurs in individuals with lower V_I/F+I/F/C_ values, i.e., with residual CFTR activity, whereas bacterial colonization is more frequent in individuals with higher V_I/F+I/F/C_ values (and absence of CFTR activity).

### V_I/F+I/F/C_ and CF Diagnosis

**FIGURE 8:**
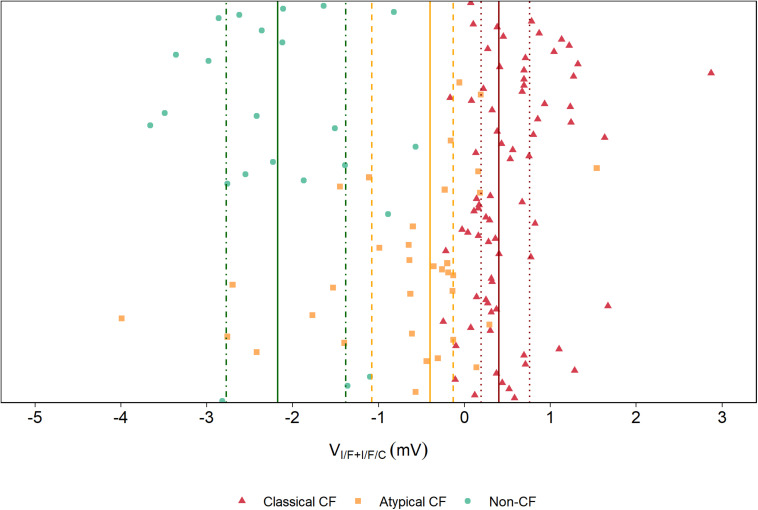
Distribution of V_I/F+I/F/C_ values in the studied cohort. A distribution showing the relative position of each individual in this study according to the new suggested parameter show that the median (line) of the V_I/F+I/F/C_ for each diagnosis group and the 25%/75% percentile (dotted line) clearly distinct Non-CF (green), Atypical CF (yellow) and Classical CF (red), and could be used to indicate whether a certain individual is more likely to have Classical CF, Atypical CF or non-CF.

Using this new parameter, it is possible to establish a threshold that distinguishes Classical CF from Atypical CF from Non-CF individuals (Figure 8), by taking the median and the 25%/75% percentile of the V_I/F+I/F/C_ values for each diagnosis group ± SD. This reinforces the idea that measuring CFTR function in rectal biopsies, along with the genotyping of CFTR and/or assessment of the clinical status, could be used to help to better diagnose a suspicious case.

## Discussion and Conclusion

The establishment of a definite CF diagnosis faces several challenges, in particular for atypical forms of CF, since there is a high variability of disease symptoms and organ involvement, which it is not just directly dependent on the CFTR genotype. Moreover, the increasing number of asymptomatic babies who are CF-screen positive with inconclusive diagnosis (CFSPID), resulting from extensive newborn CF screen programs, makes it difficult for physicians to provide adequate genetic counseling and medical care. There are also significant prognosis issues related to CFTR mutations of unknown disease liability. Accordingly, laboratory assays have been developed to assist in solving these dilemmas for diagnosis confirmation/exclusion. The SwCl test is by far the most extensively validated biomarker (Beekman et al., 2014), but additional tests, albeit only performed at some CF centres, have also contributed to clarify diagnosis/prognosis difficulties. These tests include NPD measurements of *in vivo* voltage potential resulting from transepithelial ion fluxes at the nasal mucosal. However, this assay is unreliable in subjects with acute upper respiratory tract infection, extensive nasal polyps or after prior sinus surgery, and it can be potentially modified by CF-related inflammation, which decreases the specificity and sensitivity of the assay (Beekman et al., 2014). SwCl test is sensitive to small changes in CFTR function and is able to distinguish between healthy and classical CF but is more challenging to discriminate between atypical CF and CFTR-related diseases. Also, it has been shown that SwCl, being an indirect measurement of CFTR channel activity, does not totally reflect changes in lung function (Ramsey et al., 2011). *Ex vivo* analysis of fresh rectal biopsies by Ussing chamber has been so far the best discriminant biomarker for CF diagnosis/prognosis (Mall et al., 2000; Sousa et al., 2012). This biomarker is very useful when NPD measurements are not possible, i.e., when infants are too young to allow NPD being performed or in the above-mentioned situations. It is also the only laboratory test where CFTR function is measured directly *ex vivo* in the native epithelium, being very sensitive to quantitatively assess wt- and mutant CFTR function. This leads to higher discriminant power of classical and atypical forms of CF (Beekman et al., 2014). Indeed, we have previously shown that the equivalent short-circuit current of the I/F + CCH response (I’_sc–I/F/C_) is a good discriminator not only between non-CF and CF (thus valuable for a diagnosis of CF) but also between PI (usually classical) and PS (usually atypical) forms of CF. In fact, I’_sc–I/F/C_ showed good correlations with pancreatic function, SwCl and age-stratified lung function (measured by FEV_1_) (Sousa et al., 2012).

Here, we explored additional electrophysiological parameters resulting from the analysis of Ussing chamber measurements in fresh rectal biopsies in a bigger cohort with PwCF from several nationalities. Our data show that another parameter – the voltage of the maximum activation of CFTR (V_I/F+I/F/C_) – evidenced more significant and higher correlations with several clinical CF parameters/biomarkers, including pancreatic function (70 vs. 54%), SwCl concentration (58 vs. 51%), and age-stratified lung function (32 vs. 27%) (Table 1 and Figures 3, 4). These high correlations of V_I/F+I/F/C_ with pancreatic function and SwCl remain significant even after being corrected for multiple testing. These data clearly demonstrate that V_I/F+I/F/C_ is a better biomarker than I’_sc–I/F/C_ to predict CF disease severity. A possible explanation for the better correlations of V_I/F+I/F/C_ vs. I’_sc–I/F/C_ is the fact that the rectal biopsy tissue quality can severely affect the transepithelial resistance (R_te_) measured in the Ussing chamber, and the R_te_ value is used to calculate the I_eq–sc_ by Ohm’s law. Therefore, bad tissue quality, which can result from poor bowel preparation, intestinal inflammation, or other factors, can affect the R_te_ value and therefore can create a bias in the calculated I_eq–sc_ values. Actually, poor biopsy quality represents the main challenge of this protocol and can totally impair these analyses, since it can affect tissue Rte, that results in total lack of responses to compounds, lower responses than expected and can mislead to wrong conclusions. A threshold of the median of the V_I/F+I/F/C_ values for each diagnosis group was established as reference to better predict if a suspicious case is going to have a more severe or milder prognosis. We believe that it would be relevant in the future to further increase the non-CF controls in this type of analysis, as well as to measure the SwCl, lung function and pancreatic function in these controls.

The hallmark of the clinical course of CF disease is progressive loss of lung function with eventual respiratory failure. In the context of CF, lung function is perhaps the most prominent measure of disease severity, progression, and therapeutic efficacy (Szczesniak et al., 2017). The primary spirometry result of interest in CF is FEV_1_, an index of airway obstruction that has played a critical role in both clinical care and research. Over the past 50 years, FEV_1_ decline has been associated with morbidity and mortality among PwCF (Liou et al., 2001), as well as with greater risk of pulmonary exacerbation, hospitalizations and with *P. aeruginosa* colonization (Szczesniak et al., 2017), and therefore correlations with FEV_1_, as well as with FVC, were analyzed in this work. Our data show that the previously validated parameter I’_sc–I/F/C_ evidenced a lower correlation with FEV_1_ measurements than V_I/F+I/F/C_ (27 vs. 32%) (Table 1). After age-stratification, the 3–9 years group showed a significant correlation (50%) of FEV_1_ with V_I/F+I/F/C_ (Figure 5). Notably, although being the most important clinical endpoint to measure disease severity, FEV_1_ has also technical disadvantages that can impact its readout. It is highly dependent on the individuals being tested, since it requires full cooperation between the subject and the examiner, and the results obtained will depend on technical as well as personal factors, such as temperature, type of equipment, technician, among others (Miller et al., 2005). Thus, in the large multi-national cohort analyzed here, it is expected that centre-to-centre variations in the spirometry approach can influence this correlation. Recently, lung clearance index (LCI) has emerged as a better tool than FEV_1_ to monitor lung disease for all age groups (Perrem et al., 2018). However, we did not have LCI data for the current cohort. In the future, this is undoubtedly an additional correlation to be explored.

Our results showed no strong correlation of BMI with V_I/F+I/F/C_ (Table 1 and Figure 6). However, BMI simply reflects body “size” (kg/m^2^) and does not distinguish between the major metabolically active components of body composition (fat mass and fat-free mass). Independently of BMI, several studies in PwCF have described a preferential depletion of fat-free mass, which is associated with indexes of disease severity, including reduced lung function, increased pulmonary exacerbations, and increased inflammation (Alvarez et al., 2016). Therefore, studies of body composition in CF may be more informative than BMI alone as a measure of optimal health. PwCF also experience multiple bacterial infections throughout life, namely with *Pa*, being this another hallmark of CF being, for example ∼80% of PwCF by the age of 18 chronically *Pa* infected (Goeminne et al., 2015). The presence of these bacteria usually leads to a steeper decrease in lung function and worse disease prognosis. Results shown here demonstrate that V_I/F+I/F/C_ is able to significantly discriminate individuals that had bacterial colonization from those who did not, further highlighting the importance of this biomarker to predict disease prognosis.

This work highlights the fact that the assessment of CFTR-mediated Cl^–^ secretion in rectal biopsies is a reproducible technique, that can measure CFTR function directly *ex vivo* in the native epithelium, being very sensitive to quantitatively assess wt- and mutant CFTR function. Variability was observed among PwCF in different diagnostic groups, also in the same diagnostic group, between individuals with the same mutations and even between samples of the same individual. However, different degrees of variability occur in these comparisons. Among the different diagnostic groups, we observe a clear separation between non-CF (with a median Vte of ∼−2.17 mV), Atypical CF (with a median Vte of −0.4 mV), and Classical CF (with a median Vte of 0.4 mV). However, intragroup variability is also observed, especially in the Atypical CF group, which might be explained by their heterogeneity of symptoms that can reflect different degrees of CFTR dysfunction. Variation among PwCF with the same mutations is minor and they are always in the same diagnostic group. Intra- individual variability is observed and is around 5–10%.

To conclude, the establishment of a definite CF diagnosis is still challenging, particularly in individuals with atypical forms of CF. A novel parameter obtained from CFTR-mediated Cl**^–^** secretion measurements in rectal biopsies – V_I/F_**_+_**_I/F/C_ – was demonstrated here to be highly sensitive and robust for the diagnosis and prognosis of CF, evidencing good correlations with clinical parameters that indicate and predict disease liability. This is shown to be a good parameter to discriminate between classical and atypical forms of CF, one of the greatest challenges in the currently diagnosing of CF. It is shown that electrophysiological measurements in rectal biopsies *ex vivo* can improve the establishment or exclusion of a final diagnosis of CF, as well as to direct the physicians to a better disease management.

## Data Availability Statement

The original contributions presented in the study are included in the article/Supplementary Material, further inquiries can be directed to the corresponding author.

## Ethics Statement

The study was conducted in accordance with the Declaration of Helsinki, and the protocol was approved by the Ethics Committee of Research of the Faculty of Medical Sciences, University of Campinas, as well as of the Santa Maria Hospital, Lisbon, Portugal and the Paediatrics Department of Motol University Hospital, Prague, Czechia.

## Author Contributions

IS and MDA: conceptualization. IS, AD, FM, RC, and TD: methodology. IS, AD, RC, and FM: formal analysis. IS, RC, and TD: validation. IS: investigation and writing – original draft preparation. MDA and KK: resources and supervision. MDA, FM, and KK: writing – review and editing. MDA: funding acquisition and project administration. All authors have read and agreed to the published version of the manuscript.

## Conflict of Interest

The authors declare that the research was conducted in the absence of any commercial or financial relationships that could be construed as a potential conflict of interest.

## Footnotes

1 www.cftr2.org
