# Supplementary figures and images for: Assessment of Distinct Electrophysiological Parameters in Rectal Biopsies for the Choice of the Best Diagnosis/Prognosis Biomarkers for Cystic Fibrosis
Source: Front Physiol. 2020 Dec 23;11:604580. doi: 10.3389/fphys.2020.604580 (PMC7786280; doi:10.3389/fphys.2020.604580)

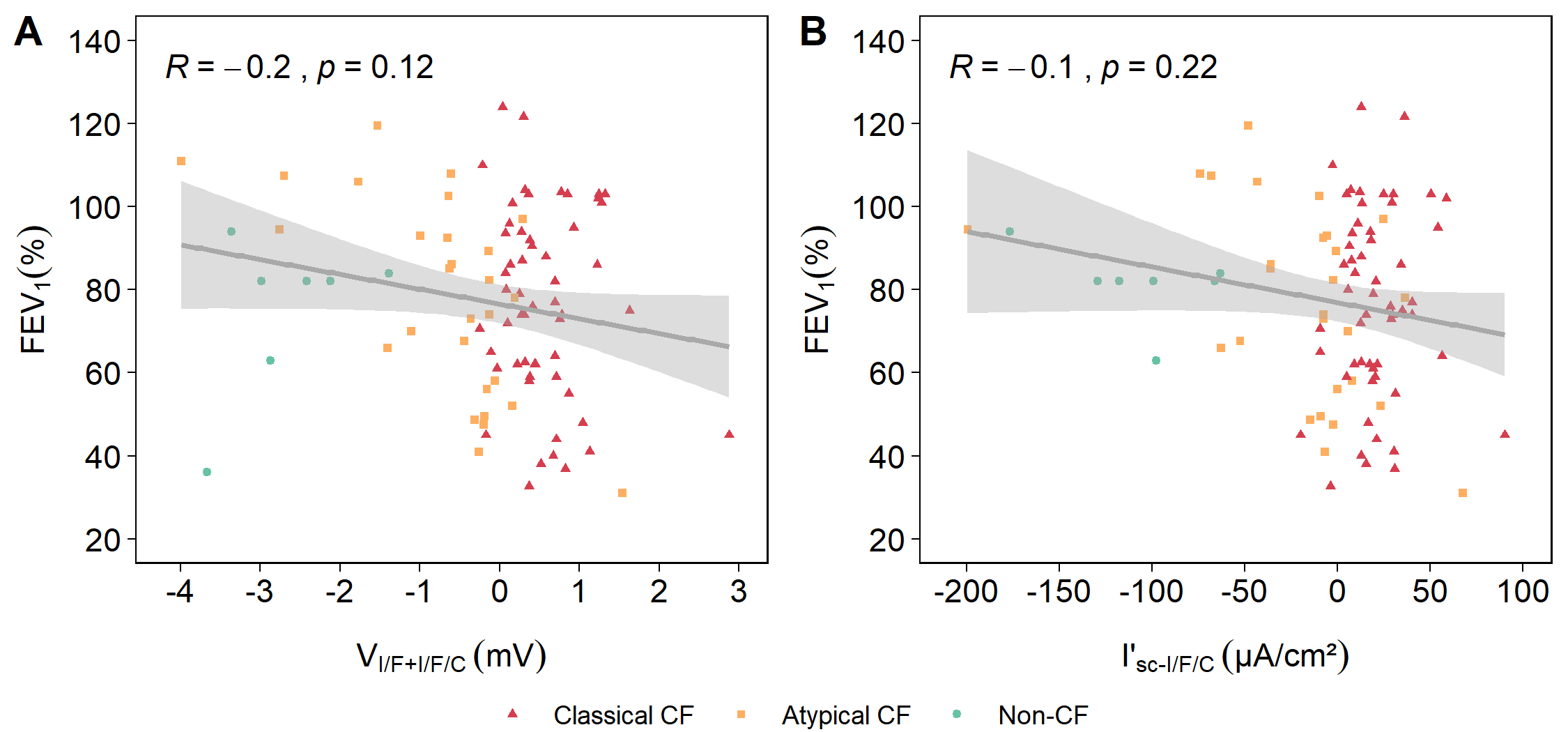

Supplement: Supplementary file 1 [file Image_1.TIFF]
