# Supplementary material for: Assessment of Distinct Electrophysiological Parameters in Rectal Biopsies for the Choice of the Best Diagnosis/Prognosis Biomarkers for Cystic Fibrosis
Source: Front Physiol. 2020 Dec 23;11:604580. doi: 10.3389/fphys.2020.604580 (PMC7786280; doi:10.3389/fphys.2020.604580)
Supplement: Supplementary file 2 [file Table_1.docx]

Supplementary Material

**Table S1** - Descriptive data regarding the CFTR genotypes, from all participants enrolled in the study.

|  | Patient code | Genotype | Diagnosis |
| --- | --- | --- | --- |
| Brazil | BRA01 | wt | Non-CF |
|  | BRA02 | wt | Non-CF |
|  | BRA03 | F508del/G542X | Classical CF |
|  | BRA04 | F508del/R1066C | Classical CF |
|  | BRA05 | W1282X/NI | Atypical CF |
|  | BRA06 | F508del/F508del | Classical CF |
|  | BRA07 | wt | Non-CF |
|  | BRA08 | F508del/S4X | Classical CF |
|  | BRA09 | wt | Non-CF |
|  | BRA10 | F508del/R334W | Classical CF |
|  | BRA11 | wt | Non-CF |
|  | BRA12 | F508del/F508del | Classical CF |
|  | BRA13 | F508del/F508del | Classical CF |
|  | BRA14 | F508del/S549R | Atypical CF |
|  | BRA15 | F508del/G542X | Classical CF |
|  | BRA16 | wt | Non-CF |
|  | BRA17 | wt | Non-CF |
|  | BRA18 | 3120+1G>A/L206W | Atypical CF |
|  | BRA19 | R1162X/NI | Non-CF |
|  | BRA20 | F508del/G85E | Atypical CF |
|  | BRA21 | F508del/3272-26A>G | Atypical CF |
|  | BRA22 | wt | Non-CF |
|  | BRA23 | wt | Non-CF |
|  | BRA24 | F508del/F508del | Classical CF |
|  | BRA25 | F508del/F508del | Classical CF |
|  | BRA26 | 3120+1G>A/R1066C | Classical CF |
|  | BRA27 | F508del/F508del | Classical CF |
|  | BRA28 | F508del/F508del | Classical CF |
|  | BRA29 | wt | Non-CF |
|  | BRA30 | F508del/F508del | Classical CF |
|  | BRA31 | F508del/R334W | Atypical CF |
|  | BRA32 | F508del/F508del | Classical CF |
|  | BRA33 | F508del/F508del | Classical CF |
|  | BRA34 | F508del/F508del | Classical CF |
|  | BRA35 | wt | Non-CF |
|  | BRA36 | wt | Non-CF |
|  | BRA37 | wt | Non-CF |
|  | BRA38 | F508del/F508del | Classical CF |
|  | BRA39 | F508del/F508del | Classical CF |
|  | BRA40 | wt | Non-CF |
|  | BRA41 | wt | Non-CF |
|  | BRA42 | F508del/F508del | Classical CF |
|  | BRA43 | F508del/1717-1G>A | Classical CF |
|  | BRA44 | F508del/1812-1G>A | Classical CF |
|  | BRA45 | F508del/F508del | Classical CF |
|  | BRA46 | F508del/R553X | Classical CF |
|  | BRA47 | wt | Non-CF |
|  | BRA48 | G542X/I618T | Atypical CF |
|  | BRA49 | F508del/F508del | Classical CF |
|  | BRA50 | F508del/G542X | Classical CF |
|  | BRA51 | F508del/F508del | Classical CF |
|  | BRA52 | F508del/P205S | Atypical CF |
|  | BRA53 | 3120+1G>A/R1066C | Classical CF |
|  | BRA54 | F508del/F508del | Classical CF |
|  | BRA55 | wt | Non-CF |
|  | BRA56 | F508del/F508del | Classical CF |
|  | BRA57 | F508del/F508del | Classical CF |
|  | BRA58 | F508del/F508del | Classical CF |
|  | BRA59 | F508del/G542X | Classical CF |
|  | BRA60 | wt | Non-CF |
|  | BRA61 | F508del/F508del | Classical CF |
|  | BRA62 | wt | Non-CF |
|  | BRA63 | F508del/F508del | Classical CF |
|  | BRA64 | F508del/F508del | Classical CF |
|  | BRA65 | wt | Non-CF |
|  | BRA66 | F508del/F508del | Classical CF |
|  | BRA67 | wt | Non-CF |
|  | BRA68 | F508del/N1303K | Classical CF |
|  | BRA69 | F508del/F508del | Classical CF |
|  | BRA70 | F508del/F508del | Classical CF |
|  | BRA71 | wt | Non-CF |
|  | BRA72 | F508del/G542X | Classical CF |
|  | BRA73 | F508del/F508del | Classical CF |
|  | BRA74 | F508del/F508del | Classical CF |
|  | BRA75 | wt | Non-CF |
|  | BRA76 | wt | Non-CF |
|  | BRA77 | F508del/F508del | Classical CF |
| Prague | CZE01 | wt | Non-CF |
|  | CZE02 | wt | Non-CF |
|  | CZE03 | wt | Non-CF |
|  | CZE04 | wt | Non-CF |
|  | CZE05 | wt | Non-CF |
|  | CZE06 | wt | Non-CF |
|  | CZE07 | wt | Non-CF |
|  | CZE08 | F508del/D1152H | Atypical CF |
|  | CZE09 | F508del/D1152H | Atypical CF |
|  | CZE10 | 2789+5G>A/F508del | Atypical CF |
|  | CZE11 | 3849+10kbC>T/F508del | Classical CF |
|  | CZE12 | 3849+10kbC>T/F508del | Classical CF |
|  | CZE13 | F508del/3272-26A>G | Atypical CF |
|  | CZE14 | F508del/F508del | Classical CF |
|  | CZE15 | F508del/D1152H | Atypical CF |
|  | CZE16 | F508del/1717-2A>G | Atypical CF |
|  | CZE17 | 2789+5G>A/F508del | Atypical CF |
|  | CZE18 | 3849+10kbC>T/dele2,3 | Atypical CF |
|  | CZE19 | N1303K/D1152H | Atypical CF |
|  | CZE20 | N1303K/D1152H | Atypical CF |
|  | CZE21 | F508del/G542X | Classical CF |
|  | CZE22 | F508del/G542X | Classical CF |
|  | CZE23 | F508del/R374P | Atypical CF |
|  | CZE24 | 2789+5G>A/F508del | Atypical CF |
|  | CZE25 | 3849+10kbC>T/621+1G>T | Atypical CF |
|  | CZE26 | 3849+10kbC>T/F508del | Atypical CF |
|  | CZE27 | 3849+10kbC>T/F508del | Atypical CF |
|  | CZE28 | F508del/S955P | Atypical CF |
|  | CZE29 | 711+5G>A/F508del | Classical CF |
|  | CZE30 | F508del/G542X | Classical CF |
|  | CZE31 | 2789+5G>A/dele2,3 | Classical CF |
|  | CZE32 | W57G/3272-26A>G | Atypical CF |
|  | CZE33 | 3272-26A>G/F508del | Atypical CF |
|  | CZE34 | F508del/F508del | Classical CF |
|  | CZE35 | F508del/F508del | Classical CF |
| Lisbon | LIS01 | V201M/NI | Non-CF |
|  | LIS02 | Y1092X/P205S | Atypical CF |
|  | LIS03 | Y1092X/P205S | Atypical CF |
|  | LIS04 | F508del/Ni | Classical CF |
|  | LIS05 | F508del/N1303K | Classical CF |
|  | LIS06 | F508del/F508del | Classical CF |
|  | LIS07 | F508del/N1303K | Classical CF |
|  | LIS08 | G85E/1717-1G>A | Classical CF |
|  | LIS09 | 3120+1G>A/3120+1G>A | Classical CF |
|  | LIS10 | F508del/F508del | Classical CF |
|  | LIS11 | F508del/D614G | Atypical CF |
|  | LIS12 | F508del/A561E | Classical CF |
|  | LIS13 | F508del/R334W | Classical CF |
|  | LIS14 | F508del/F508del | Classical CF |
|  | LIS15 | F508del/R334W | Atypical CF |
|  | LIS16 | F508del/711+1G>T | Classical CF |
|  | LIS17 | G85E/NI | Classical CF |
|  | LIS18 | N1303K/N1303K | Classical CF |
|  | LIS19 | R75Q/NI | Non-CF |
|  | LIS20 | G85E/G85E | Classical CF |
|  | LIS21 | A561E/A561E | Classical CF |
|  | LIS22 | R1162X/R334W | Classical CF |
|  | LIS23 | G542X/Q220X | Classical CF |
|  | LIS24 | G542X/R334W | Classical CF |
|  | LIS25 | A561E/A561E | Classical CF |
|  | LIS26 | Y1092X/R334W | Atypical CF |
|  | LIS27 | 3272-26A>G/3171delC | Atypical CF |
|  | LIS28 | I148N/P205S | Atypical CF |
|  | LIS29 | I148N/P205S | Atypical CF |
|  | LIS30 | Q1100P/S4X | Classical CF |
|  | LIS31 | 3272-26A>G/3299A>C | Classical CF |

WT, wild-type; NI, not informed; CF, cystic fibrosis.

**Table S2** - Descriptive data regarding the demographical, clinical and laboratorial markers with categorical distribution from all participants enrolled in the study.

| **Parameters** |  | **Data distribution [n/N (%)]** |
| --- | --- | --- |
| Gender | Female | 70/143 (49) |
|  | Male | 73/143 (51) |
| Diagnosis group | Classical CF | 75/143 (52) |
|  | Atypical CF | 34/143 (24) |
|  | Non-CF | 34/143 (24) |
| Bacterial colonization | Present | 75/106 (71) |
| *Pseudomonas aeruginosa* | Present | 49 /106 (46) |
| Pancreatic insufficiency | Present | 59/95 (62) |
| SwCl (mEq/L) | < 30 | 5/119 (4) |
|  | 30 to 60 | 16/119 (13) |
|  | > 60 | 98/119 (82) |
| FEV_1_ (%) | < 80 | 48 /98 (49) |
|  | ≥ 80 | 50 /98 (51) |

N, number of individuals; %, percentage; SwCl, sweat chloride; mEq/L, milliequivalent per litre; FEV_1_, forced expiratory volume in the first second of the forced vital.

**Table S3** – Descriptive data regarding the demographical, clinical and laboratorial markers from the participants enrolled in the study per group.

| **Patient condition** | **Parameter** | **N** | **Mean ± SD** | **Median** | **Percentile** | **95% CI** |
| --- | --- | --- | --- | --- | --- | --- |
| Classical CF | Age (years) | 74 | 13.95 ± 10.7 | 11 | 8 to 17 | 11.47 to 16.42 |
|  | SwCl (mEq/L) | 72 | 109.76 ± 21.52 | 108.9 | 97.25 to 124.1 | 104.7 to 114.82 |
|  | FEV_1_ (%) | 58 | 76.08 ± 22.73 | 75.5 | 61.25 to 93.92 | 70.11 to 82.06 |
|  | FVC (%) | 55 | 84.72 ± 20.42 | 87 | 74.5 to 97 | 79.2 to 90.24 |
|  | BMI (Kg/m^2^) | 64 | 18.23 ± 3.88 | 17.25 | 15.01 to 21 | 17.26 to 19.2 |
|  | FE-1 (μg/g) | 59 | 82.52 ± 298.54 | 10 | 5.34 to 15.3 | 4.72 to 160.32 |
| Atypical CF | Age (years) | 34 | 19.26 ± 14.7 | 16 | 7 to 33.5 | 14.14 to 24.39 |
|  | SwCl (mEq/L) | 34 | 77.62 ± 33.47 | 84 | 53.84 to 96.39 | 65.94 to 89.3 |
|  | FEV_1_ (%) | 30 | 80.22 ± 24.43 | 83.72 | 60 to 101.12 | 71.1 to 89.34 |
|  | FVC (%) | 29 | 85.6 ± 17.4 | 89.3 | 83 to 95 | 78.98 to 92.21 |
|  | BMI (Kg/m^2^) | 31 | 18.81 ± 4.4 | 18 | 16.14 to 20.43 | 17.19 to 20.42 |
|  | FE-1 (μg/g) | 29 | 675.26 ± 490.05 | 516 | 433 to 900 | 488.86 to 861.67 |
| Non-CF | Age (years) | 33 | 18.18 ± 18.27 | 13 | 8 to 19 | 11.7 to 24.66 |
|  | SwCl (mEq/L) | 13 | 52.55 ± 19.23 | 46 | 42 to 66.39 | 40.92 to 64.17 |
|  | FEV_1_ (%) | 10 | 83.2 ± 19.44 | 83 | 82 to 91.75 | 69.3 to 97.1 |
|  | FVC (%) | 10 | 94.4 ± 19.1 | 94 | 90.5 to 103.75 | 80.73 to 108.07 |
|  | BMI (Kg/m^2^) | 12 | 20.83 ± 5.85 | 20.4 | 15.05 to 25.72 | 17.12 to 24.55 |
|  | FE-1 (μg/g) | 7 | 506.68 ± 238.35 | 524.25 | 465.82 to 656.13 | 286.24 to 727.11 |

N, number of individuals; %, percentage; BMI, body mass index; SwCl, sweat chloride; mEq/L, milliequivalent per litre; FE-1, faecal elastase E1; FEV_1_, forced expiratory volume in the first second of the forced vital; FVC, forced vital capacity; SD, standard deviation; CI, confidence interval.


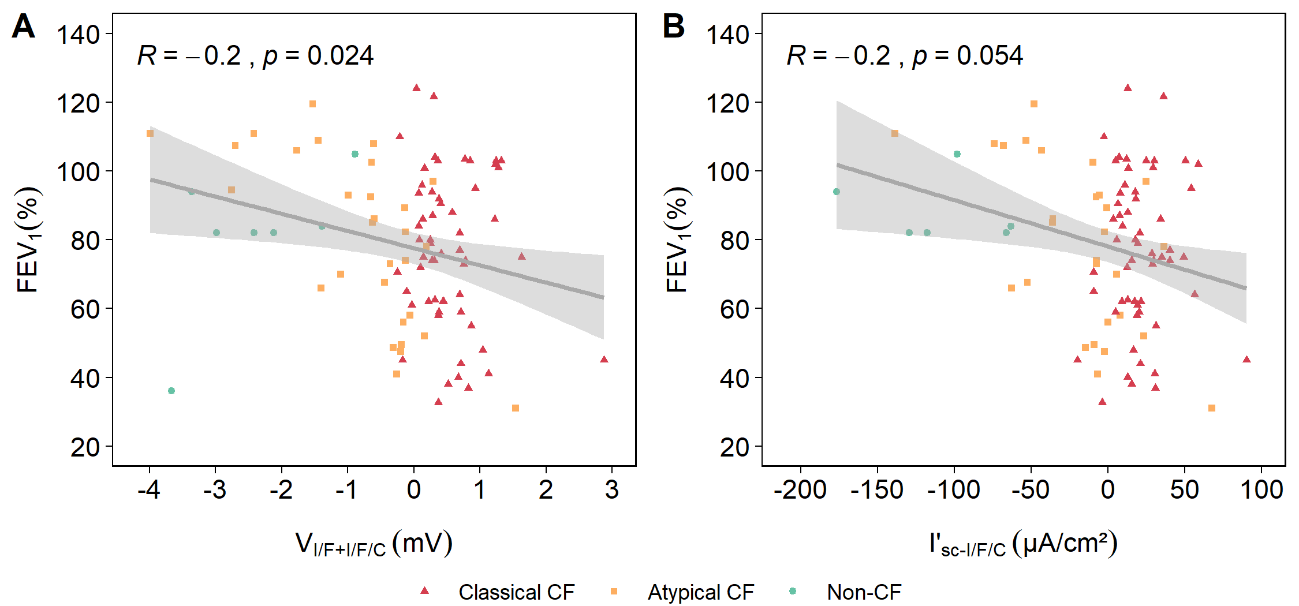


**Figure S1 – Correlation between forced expiratory volume in 1 second (FEV_1_ (%)) and V_I/F+I/F/C_** **and I’_sc-I/F/C_.** Spearman rho rank correlation analysis showed that there is no correlation between FEV1 (%) and **(A)** V_I/F+I/F/C_ nor **(B)** I’_sc-I/F/C_ when all individuals are pooled together.
